# Half-castration is a newly effective method for increasing yield and tenderness of male cattle meat

**DOI:** 10.5713/ab.21.0536

**Published:** 2022-01-21

**Authors:** Van-Ba Hoa, Dong-Heon Song, Kuk-Hwan Seol, Sun-Moon Kang, Hyun-Wook Kim, Sun-Sik Jang, Soo-Hyun Cho

**Affiliations:** 1 Animal Products Utilization Division, National Institute of Animal Science, RDA, Wanju 55365, Korea; 2 Hanwoo Research Institute, National Institute of Animal Science, Pyeongchang, 25340, Korea

**Keywords:** Castration, Half-castration, Meat Quality, Meat Yield, Tenderness

## Abstract

**Objective:**

For improving meat quality especially tenderness, male cattle are usually castrated to removes both the testicles. This study was conducted to evaluate the effect castration method (half- and complete-castration) on meat yield and quality characteristics of Hanwoo male cattle.

**Methods:**

Thirty-two similar age (5.9 months) Hanwoo male calves were divided into: half-castration (HC) and complete-castration (CC) groups (n = 16 per group). At 7 months of age, all the animals were castrated in which the HC calves had only one testicle surgically removed while, the CC calves had both testicles surgically removed. The castrated animals were reared under identical conditions until 25 months of age. After slaughter, the carcasses were evaluated for carcass traits and meat yield of primal cuts. For examination of the castration effect on meat quality, *L. lumborum* and *semimembranosus* muscles were used. The meat samples were analyzed for chemical composition, color, pH, shear force and water holding capacity, fatty acids, metabolites and volatile aroma compounds.

**Results:**

The HC group showed higher meat yields of all primal cuts (p<0.05). As a result, the total meat yield was higher by approximately 44 kg in the HC group (303.32 kg, corresponding to 67.88%) compared to the CC group (259.30 kg, corresponding to 62.11%) (p< 0.05). In terms of meat quality, the HC resulted in two times greater fat content in both muscles examined compared to intact males. More importantly, the shear force values did not differ between HC and CC groups for *L. lumborum* muscles (p>0.05). The meat from HC animals exhibited higher amount of free amino acids associated with sweetness (p< 0.05). Furthermore, the castration method only exhibited a negligible effect on metabolites and volatile aroma compounds in the cooked meat.

**Conclusion:**

Half-castration emerged as an alternative practice to be used for increasing the yield and tenderness of male cattle meat.

## INTRODUCTION

Meeting the quality and quantity demands for consumers is of vital importance for the beef industry. Despite the Corona virus pandemic disruptions, consumer demand for beef still remained strong in 2020, with per capita consumption of about 26.53 kg [1]. However, not only did consumers consume more beef, they also requested higher-quality beef [2] due to the changes in consumer preferences and quality perceptions [3]. Consumer studies have shown that the preference and purchasing decision for beef rely on a number of factors such as lean color, marbling (intramuscular fat [IMF]) degree and tenderness etc. which also vary among the markets [4]. In general, almost all consumers consider meat tenderness as the important qualitative trait that determines acceptability, satisfaction and willingness-to-pay premium prices [5]. In some markets, beside the tenderness, the marbling (IMF) degree is the most important factor determining the overall eating quality, purchasing decision and price of beef [4]. Because, the marbling level has been found to be the main driver of beef eating quality as it positively affects tenderness, juiciness, flavor and overall liking [6]. Contrastingly, in some countries, a significant ratio of consumers generally do not prefer highly-marbled beef due to the health concerns [7]. In this context, producing tenderness-guaranteed beef with different IMF levels (e.g., low, moderate and medium) is necessary to meet the demands of these market segmentations.

Pre- or post-pubertal castration of male cattle is a common management practice in beef production systems. Castration is a process that removes or inactivates the testicles of a bull by using either surgical or stopping blood flow to the testes and immunocastration methods [8]. The castration has been reported to produce a lot of advantages such as decreased aggressiveness and sexual behavior, enhanced on-farm safety and stopped male hormone production etc. [8,9]. However, the castration also has some major disadvantages such as slower growth rate and lesser feed conversion efficiency compared to non-castration (intact males) [10,11]. This is due to the reduced concentrations of serum growth-enhancing sexual hormones such as testosterone in the castrated males (steers) since it is mainly responsible for muscular tissue development [9,12]. Practically, the castration has been proposed as a means of enhancing meat quality especially tenderness because castration of bulls increases carcass back-fat thickness [13] and IMF content [13,14]. In countries such as Korea, for highly marbled beef meat production, approximately 98% of male calves are subjected to surgical castration to remove both testicles [15]. As above-mentioned, the growth-enhancing sexual hormones (e.g., testosterone) strongly affects growth rate and metabolisms such as myonucleic formation and protein synthesis [16], and daily weight gain [12]. Since the testosterone is mainly produced by two testes, maintaining a certain level by removing only one testicle may have a potential to improve the IMF accumulation and meat quality (tenderness) while, still supporting muscular protein synthesis.

In this study, two different surgical castration methods: i) complete castration (CC, completely removed both testicles), and ii) half-castration (HC, removed only one testicle) were applied to investigate their effects on carcass traits, meat yield and quality characteristics of Hanwoo cattle.

## MATERIALS AND METHODS

### Animal care

The animal and protocols used in the present study were reviewed and approved by the Institutional Animal Care and Use Committee (IACUC) at National Institute of Animal Science (Approval No. NIAS 20001992).

### Animals and samples preparation

In the present investigation, the trials were carried out on 32 similar age (5.9 months) Hanwoo male calves. The calves were randomly divided into i) HC (initial body weight: 147.53 kg) and ii) CC (initial body weight: 149.33 kg) groups (n = 16 per group). At 7 months of age, all the animals were castrated in which the HC calves had only one testicle surgically removed while, the CC calves had both testicles surgically removed. Following the castration, the castrated animals were reared in separated pens (4 calves/pen with an area of 5×10 m^2^) under the identical environmental condition and fed the same diet. Particularly, during the growing period (6 to 14 months of age), approximately 3.0 to 7.5 kg of formula feed and 3 to 4.0 kg of Italian ryegrass per animal were provided. At the fattening period (15 to 25 months of age), approximately 8 to 9.5 kg of formula feed and 1 to 3.0 kg of rice straw per animal were provided. The details on ingredients and chemical composition of experimental diets are described in our previous study [17]. At the end of fattening period, the animals were shipped to a practical plant (Jeonju, Korea) where they were slaughtered following the commercial procedure, and the carcass sides were chilled at 2°C in a chilling room. Twenty-four hours after slaughter, the carcasses were evaluated for carcass traits (e.g., weight and dressing), and then they were fabricated into primal cuts that were deboned and trimmed of external fat and connective tissues for meat yield determination following the Korean Hanwoo Beef Specification Guide [18].

For examination of the effect of castration method on meat quality, two representative muscles (*L. lumborum* and *semimembranosus*) were used. The meat samples were then cut into sub-samples depending on analyses in which the chemical composition, color, shear force, pH and water holding capacity (WHC) were performed on fresh sub-samples (the sampling day), and vacuum packed and storage frozen (−20°C) sub-samples were used for analysis of fatty acids, metabolites, and volatile aroma compounds.

### Proximate composition

The moisture, fat, protein, and collagen contents were analyzed following the AOAC Official Methods 2007.04 by using a Food Scan Lab 78810 (Foss Tecator Co., Ltd., Hillerod, Denmark). Briefly, about 200 g of ground meat sample (each) was distributed onto the round dish which was then loaded into the instrument’s sample chamber. Based on the pre-calibrated ranges of moisture, protein, fat, and collagen for meat category by manufacturer, these contents were automatically measured and expressed as percentage. Each sample was determined in duplicate.

### pH measurement

The pH values of samples were measured in triplicate using a pH*K 21 meter (NWK-Technology GmbH, Kaufering, Germany) equipped with a stainless steel and solid-state probe. The pH measurement was done by inserting the probe deeply into the muscle tissues. Prior to use, the pH meter was calibrated with pH 4.0 and 7.0 standard solutions (NWK Technology., Germany).

### Meat color and Warner-Bratzler shear force

The color and Warner-Bratzler shear force (WBSF) were measured on a same transverse section (3.0 cm-thick steak) of each the sample. For the color, after a 30 min blooming period at 4°C, it was measured on three different locations of each sample using a standardized Minolta Chroma Meter CR-400 with a D65 illuminant*C and 2° observer (Minolta Camera Co, Osaka, Japan). The color was measured under white lighting and the results were reported as CIE L* (lightness), CIE a* (redness), and CIE b* (yellowness).

For the WBSF analysis, it was carried out following our previous procedure [19]. The samples were placed into individual plastic bags, sealed with double clip, and placed in a 72°C pre-heated water bath until the core temperature reached 70°C. Immediately after cooking, the cooked samples were cooled for 30 min under running water and each sample was made into 5 strips parallel to the muscle fiber direction using a 0.5-inch metal corer. The WBSF values were obtained by completely cutting the strips using a Instron Universal Testing Machine (Model 4465, Instron Corp, High Wycombe, UK) at a crosshead speed of 200 mm/min and a 40 N load cell.

### Fatty acid composition

The sample preparation for fatty acids analysis was performed following the procedure as described in our previous study [19]. Briefly, each sample (Ca.10 g) was weighed and homogenized with 150 mL of chloroform: methanol (2:1, v/v) solvent mixture at 300×g for 3 min using a homogenizer (Polytron, PT-MRC 2100, Littau, Switzerland). After filtration through Whatman filter paper, the filtrate was added with approximately 20 g of Na_2_SO_4_, thoroughly mixed for 1 min, and then the upper lipid layer was transferred into an Erlenmeyer flask. After drying at 55°C using a rotary evaporator, the lipids layer was reconstituted with 1 mL tricosanoic acid and 1 mL of 0.5 N NaOH. Finally, the lipid was converted to fatty acid methyl esters (FAMEs). Approximately 1.0 mL of FAMEs was taken and placed into auto-sampler vials, sealed, and used for fatty acids analysis. The separation of FAMEs was achieved using a gas chromatography/flame ionization detector (GC-FID; Varian Technologies, Palo Alto, CA, USA) equipped with an Omegawax capillary column (30 m×0.25 mm×0.25 μm film thickness; Supelco, Bellefonte, PA, USA) as described in our previous study [19]. Identification of fatty acids in the samples was carried out by comparing their retention times with those obtained from standard fatty acids. Individual fatty acids were expressed as relative percent (%) of total fatty acids.

### Metabolites analysis by NMR Spectroscopy

The analysis of metabolites was performed using the protocol as described in our previous study [20] with suitable modification. Approximately 20 mg of each sample was weighted and extracted with acetonitrile/water (1:1, v/v) mixture on ice for 10 min. The samples were centrifuged at 3,000×g for 10 min at 4°C, and the supernatant was collected and freeze-dried. The lyophilized samples were dissolved in 700 μL of deuterated water containing 2 mM 3-trimethylsilyl-2,2,3,3-tetradeuteropropionicacid-d4 (TSP-d4; Sigma-Aldrich, St. Louis, MO, USA) as an internal reference, and then was transferred into a 5 mm NMR tube for analysis. ^1^H-NMR spectra were acquired on a 600 MHz Agilent NMR spectrometer (Agilent Technologies, Palo Alto, CA, USA) equipped with 600 MHz 4-mm gHX NanoProbe (Agilent Technologies, Santa Clara, CA, USA) at a ^1^H frequency of 599.93 MHz. The ^1^H-NMR conditions set were the same as those described in our previous study [20]. The acquired spectra were phased and then the baseline was corrected and referenced to the TSP-d4 peak using a Vnmrj (version 4.2, Agilent Technologies, USA). Metabolites in the ^1^H-NMR spectra were tentatively identified using Chenomx 600 MHz library database and Chenomx NMR Suite 7.1 professional (Chenomx Inc., Edmonton, Canada). The identified metabolites were expressed as percentage of the normalized area.

### Free amino acids and nucleotides analysis

The free amino acids (FAAs) content in the samples was analyzed following the procedure as described by Cho et al [20] with suitable modifications. Briefly, 5.0 g of each sample was weighted and homogenized with 10 mL distilled water at 11,000×g for 30 s. After centrifuging at 10,000×*g* for 10 min at 4°C, 100 μL of supernatant was taken and mixed with 900 μL methanol containing 0.1% formic acid. The samples were again centrifuged at 10,000×*g* for 10 min at 4°C, and about 500 μL of supernatant was injected into a Waters ACQUITY UPLC (model: Xevo TQ-S; Waters Co. Milford, MA, USA) connected to an Imtaka Intrada Amino Acid column: 2×50 mm, 3 μm (Imtaka, Uphur St, Suite A, Portland). The eluents used were A (acetonitrile: 100 mM ammonium formate; 20:80 v/v) and B (acetonitrile: trifluoroacetic acid: 25 mM ammonium formate: formic acid: 9:75:16:03 v/v/v). The separation was carried out at 37°C and flowing rate of 0.4 mL/min, and solvent gradient: initial 100% B, linear change to 83% B for 6.5 min, linear change to 100% A for 3.5 min and then linear change to 100% B for 2 min, and maintained for additional 5 min. The amino acids standard was used for identifying and quantifying the FAA in the samples, and the detected FAA were expressed as milligram per 100 g meat (mg/100 g meat).

### Volatile aroma compounds analysis

Volatile aroma compounds in cooked meat samples were analyzed using the procedure as described in our previous study [21]. The samples were cooked at around 180°C on an open tin-coated grill for about 2 min, and proximately 2.0 g of each the cooked samples were weighed, placed into 20-mL headspace vial (Agilent, USA) and tightly capped with PTFE-faced silicone septum prior to the analysis. The volatile compounds were extracted using solid-phase micro-extraction (SPME) technique. The vials containing samples were extracted for volatile compounds using the SPME technique. For this, a SPME device with carboxen–polydimethylsiloxane (75 μm) fiber (Supelco, USA) was inserted into the vials and held for 50 min at 60°C. All the extraction steps were carried out using an SPME auto-sampler (model: PAL RSI 85; Agilent Technologies, Santa Clara, CA, USA) connected to a gas chromatography (model: 8890 GC system; Agilent Technologies, USA) with mass spectrophotometry (5977B MSD; Agilent Technologies, USA). Immediately after the extraction, the fiber was desorbed for 5 min at 250°C, and the compounds were separated on a DB-5MS capillary column (30 m×0.25 mm i.d.×0.25 μm film thickness: Agilent J & W Scientific, Folcom, CA, USA) using helium as a carrier gas. The GC oven was programmed to 40°C for 5 min and then increased to 250°C at a rate of 8°C/min and held at this temperature for further 5 min. The MS conditions set were capillary direct interface temperature, 250°C, scanning mass range of 30 to 500 amu and rate at 5.27 scans/s. The volatile compounds were identified by using the Wiley registry library (Agilent Technologies, USA) and/or by external standards. The qualification of the identified compounds was done by using a concentration-known internal standard (1.0 μL of 2-methyl-3-heptanone, 816 mg/mL in methanol).

### Statistical analysis

The statistical analysis system (SAS) package (SAS Institute, Cary, NC, USA, 2015) was applied for analysis of the data. The data were analyzed by using the General Linear Model procedure of the SAS, and the castration method was considered as the main effect while the quality traits examined were considered random in the model. Means were compared using Duncan’s multiple range test. Significance was set at p<0.05. Principal component analysis (PCA) was also used to explore relationships between the variables (meat and fat yield and selected quality traits) and castration groups using the XLSTAT program 2020.3 (Addinsoft Inc., NY, USA). In this PCA model, the data obtained from both the *L. lumborum* and *semimembranosus* muscles in each castration group were averaged for each the selected variables.

## RESULTS AND DISCUSSION

### Effects on meat yield

The effect of castration on the meat yield of primal cuts are shown in Table 1. As expected, though the slaughter weight did not differ between the two groups the meat yield (weight and percentage) of all primal cuts (except the rib) were significantly (p<0.05) higher in the HC group compared to the CC group. As a result, the total meat yield was significantly higher in the HC group (303.32 kg, corresponding to 67.88%) compared to the CC group (259.30 kg, corresponding to 62.11%) (p<0.05). Thus, the total meat yield of the HC groups was about 44 kg more than the CC group. Contrastingly, a significantly higher trimmed fat level (weight and yield percentage) was found in almost all primal cuts from the CC group compared to the HC group (p<0.05). The total trimmed fat in the CC group was about 34 kg more than the HC group, indicating a higher fat deposition level in these completely castrated animals. In the present study, the animals were raised under identical conditions and fed a same dietary regime, therefore, the results indicating the higher meat yield and lower trimmed fat amount in the HC group could be attributed to its higher residual serum testosterone levels (the serum testosterone level remained in the HC group was around 10 ng/mL throughout the trial period whereas, it remained less than 1.0 ng/mL in the CC group, data not shown). Because testosterone promotes the muscular development by interacting with receptors in DNA which results in an increased protein synthesis [16]. This finding agrees well with that of Marti et al [22], who showed a positive correlation between the residual serum testosterone level and body weight gain of immunologically castrated cattle. Thus, it may be said that half-castration could be considered as an alternative practice for increasing the meat yield of castrated animals in the beef industry.

### Effects on chemical composition and quality traits

The chemical composition and quality traits of two muscles as affected by castration and their comparison with intact males (bulls) are shown in Table 2. In both the muscles examined, the HC animals had significantly (p<0.05) higher protein contents compared to the CC animals. However, when compared to the protein content in intact males, these protein levels were lower (p<0.05). This could be related to the higher serum testosterone level which increased the muscular protein synthesis in these half-castrated animals [16]. IMF (fat present within the lean) is known as the important factor determining overall meat quality [6]. Our results showed that the CC group exhibited significantly higher fat content (17.53% and 10.13% in the *L. lumborum* and *semimembranosus* muscles, respectively) compared to the HC group (8.02% and 4.28% in the *L. lumborum* and *semimembranosus* muscles, respectively) (p<0.05). Especially, the fat levels in the muscles of HC animals were almost two times greater compared to those of intact males (p<0.05). In general, the fat contents in both muscles were in the following order: CC>HC>intact males. This indicates that not only the complete castration, but the half-castration method also caused an increased degree of fat deposition in the muscle tissues when compared to the bulls. The results indicating the increased IMF deposition in the castrated males could be attributed to the lower residual serum sex hormones such as testosterone [22] because this hormone is responsible for the adipose tissue distribution, adipogenesis as well as adipocyte mechanism regulations [23], especially the testosterone has an anti-adipogenic effect on the adipose tissue [24]. Compared with fat levels in *Longissimus* muscles from castrated Friesian cattle (around 4.5%) reported by Prado et al [11] and 1/2 Nelore × 1/2 Aberdeen Angus steers (3.38%) reported by das Gracas Padre et al [25] the half-castrated cattle in the present study had a higher level.

The pH and WHC is an important technological quality trait of meat. Our results showed that the pH values in both muscles did not differ between the HC and CC groups (p> 0.05). This indicated that the rate of postmortem pH decline was similar for both castration groups. Similarly, no differences in WHC occurred between the HC and CC groups (p>0.05). However, compared with the meat of intact males, both the HC- and CC-derived meat exhibited a lower WHC (p<0.05). It is well known that WHC of meat is greatly affected by pH; a slower rate of pH decline or a higher pH value results in a better WHC of the meat by creating a charge imbalance [26]. Thus, the results indicating the higher WHC of meat from intact males may be related to its higher pH value.

There has been an increasing interest in the improvement of meat tenderness by producers as it is considered as the important qualitative trait determining acceptability, satisfaction and willingness-to-pay premium prices [5]. Among the meat tenderness evaluation methods, the WBSF is a highly reliable method for objective evaluation of beef tenderness [27]. Results showed that there were no differences in the WBSF values in the *L. lumborum* muscles between the HC (5.68 kg) and CC (5.54 kg) groups (p>0.05), but in the *semimembranosus* muscle the WBSF value was high in the HC (5.92 kg) compared to CC group (5.17 kg) (p<0.05). However, compared to the WBSF values in both the muscles (7.72 and 7.02 kg for the *L. lumborum* and *semimembranosus* muscles, respectively) from the intact males, both the HC and CC animals showed significantly lower values (p<0.05). According to the classification of beef tenderness proposed by Boleman et al [28] both the muscles from the HC and CC groups could be considered as ‘moderate’ whereas, the meat from intact males could be considered as ‘tough’ (5.90 to 7.21 kg). This indicates that not only the complete castration the half-castration method also showed its beneficial effects on the improvement of meat tenderness. This could be related to the castration which resulted in the reduced physical and sexual behavior activity [9], and an increased fat content because the level fat has been found to positively correlate with the tenderness [19]. Compared with our data, Destefanis et al [29] and Amatayakul-Chantler et al [30] reported higher shear force values (7.15 to 9.07 kg) in same muscle from castrated Piemontese and *Bos indicus* cattle.

Consumers consider color as the most important indicator of freshness and wholesomeness of meat. Regarding the L* (lightness), the CC group showed higher values in the *L. lumborum* muscles (p<0.05), but no differences occurred between the two castration groups for the *semimembranosus* muscles (p>0.05). For the a* (redness), there were no differences in the values between the HC and CC groups for both *L. lumborum* and *semimembranosus* muscles (p>0.05). It is worth noting that the HC or CC castration resulted in a better color (lighter color) compared to the intact males that presented a darker color (indicated by lower L* and higher a* values) (p<0.05). This could be related to the higher fat content in these meat sample groups whereas, the darker color of intact male meat may be due to a high level of serum testosterone which results in greater connective tissue and hemoglobin contents in the meat [14].

### Effects on fatty acid profiles

Fatty acid profiles not only reflect the nutritional value but also remarkably influence the development of meat flavor during cooking [31]. This study for first time investigated the fatty acid profiles in meat from half-castrated cattle. The outcome of our analysis (Table 3) depicts that the HC group contained lower levels of saturated fatty acids (SFA) such as myristic acid (C14:0) and palmitic acid (C16:0), and higher levels of unsaturated fatty acids such as linoleic acid (C18:2n6), gamma linoleic acid(C18:3n6) and linolenic acid (C18:3n3) in the *L. lumborum* muscles (p<0.05). A similar trend was also observed in the *semimembranosus* muscles in which the HC group contained higher levels of unsaturated fatty acids such as C18:2n6, C18:3n6 and arachidonic acid (C20:4n6) compared to the CC group (p<0.05). As a result, the total polyunsaturated fatty acid (PUFA) and n-3 PUFA contents as well as PUFA/SFA value in both muscles were higher in the HC group compared to the CC group (p<0.05). In general, research conducted to examine the effects of castration on fatty acid profiles in beef have also shown that castration caused alterations of fatty acid profiles in which a lower PUFAs and a higher monounsaturated fatty acids (MUFAs) content was observed in steers compared to bulls [14,25]. Compared to our data, however, these researchers found a lower MUFAs content (39% to 42%), this could be related to the feeding diet and genetic differences among the studies.

From a human nutritional point of view, dietary n-3 PUFAs has long been known to have many beneficial effects on physiological processes, and a healthy diet should have a high PUFA/SFA and a low n-6/n-3 ratio [32]. Furthermore, MUFAs such as oleic acid (C18:1n9) plays an important role in cooked meat flavor development [33]. As aforementioned, meat of castrated males (steer) usually contains a higher MUFAs content compared to bulls [14,25]. It is worth noting that in both the muscles studied, the most dominant MUFA such as C18:1n9 that was not different between the HC and CC groups (p>0.05). Overall, it may be said that the meat from half-castrated animals were improved in term of nutritional value by increasing the PUFAs content while their MUFAs content was comparable to that of meat from the completely castrated animals (steers).

### Effects on taste–related compounds (free amino acids and metabolites)

The concentrations of FAAs in the *L. lumborum* and *semimembranosus* muscles from two castration groups are presented in Table 4. FAAs are known as amino acids that are present in meat in the free form. FAAs play an important role eliciting tastes of meat as they are rapidly detected by the protein-coupled receptors on taste cells [34]. Researchers correlated the presence of FAAs with tastes of meat such as sweetness (glycine, alanine, serine, proline and glutamic acid), umami taste (glutamic acid, aspartic acid, alanine, serine, lysine, and methionine), sourness (aspartic acid, histidine, and asparagine) and bitterness (valine, leucine, isoleucine, phenylalanine, arginine, proline, tryptophan, and methionine) [35,36]. Our results showed that the castration only affected amounts of 3 FAAs (alanine, methionine, and glutamine). Interestingly, the concentrations of alanine and glutamine (sweetness and umami-related compounds) were approximately 4 mg higher in the HC group compared to the CC group (p<0.05). Whereas the amount of methionine was higher in both the muscles from the CC group compared to the HC group (p<0.05). The most abundant FAAs we found in the muscles from both the castration groups were alanine and glutamine, which was similar to finding of Cho et al [20], who studied on the same Hanwoo beef muscle. Based on their taste contribution as above-mentioned, the total sweetness-related FAAs content was higher (by about 7 and 3 mg in the *L. lumborum* and *semimembranosus* muscles, respectively) in the HC group compared to the CC group (p<0.05). While no differences in total amounts of umami-and sourness-related FAAs occurred between the CC and HC groups (p>0.05).

A total of 30 metabolites were identified in the muscles from the two castration groups (Table 5). Out of them, creatine, carnitine, and creatinine are the commonly found metabolites in meat [37]. Some amino acids (alanine, glutamine, isoleucine, methionine, valine, and leucine), sugars (glucose and glucose-6-phosphate), nucleotides (inosine and inosine-5′-phosphate, IMP) and others, have been reported in beef [20]. Several of the identified metabolites such as amino acids (alanine, glutamine and methionine), free sugar (glucose) and IMP have been reported to directly contribute to sweet and umami tastes of meat [38]. It was also observed that only two compounds that showed differences (p<0.05) between the HC and CC groups were glucose and methionine. Overall, the half-castration method caused a negligible effect on the tastes-related components such as FAAs and metabolites in the muscles studied, suggesting that the half-castration method seems not to cause a defect or alteration of tastes of the beef.

### Effects on volatile flavor compounds

Flavor comprising two sensations (taste and aroma) is very important from the eating quality point of view [39]. As a part of the cooked meat flavor, aroma (smell) is mainly generated by volatile compounds which are produced during cooking/heating [31]. Table 6 shows the concentrations (μg/g meat) of volatile compounds in the cooked *L. lumborum* muscles from the two castration groups. In the present study, a total of forty-five volatile compounds including aldehydes (17), alcohols (7), sulfur-and nitrogen-containing compounds (4), pyrazines (5) and hydrocarbons (12) was identified. In the cooked meat samples of both the castration groups, aldehyde was the most predominant class of aroma compounds. Aldehydes associated with fatty and fruity aromas [39], are mainly produced in cooked meat from the oxidation/degradation of fatty acids, and some from the Strecker degradation of amino acids in the Maillard reaction [31]. Out of the aldehydes, 12 compounds were produced from the fatty acids degradation and only four (2-methylpentanal, 2-methylpropanal, 2- and 3-methylbutanal) were produced from the Strecker of amino acids [40]. This indicates that the meat from both castration groups is generally characterized by their high intensity of fatty aroma notes because of increased fat content [31]. The statistical analysis showed that the castration only affected three fatty acids-derived aldehydes (hexanal, octanal and E,2-decenal) whose amounts were higher in the HC group compared to the CC group (p<0.05). The higher amount of these long-chain aldehydes (hexanal and E,2-decenal) may be related to the higher level of C18:2n6 content in the HC group (Table 3), since this fatty acid is the main source for generation of the aldehydes during cooking [40]. Out of the alcohols, only 1-octen-3-ol was affected by the castration, with a higher amount found in the HC group (p<0.05). Furthermore, sulfur-and nitrogen-containing compounds as well as pyrazines associated with meaty and roasted aroma notes are important in the cooked meat flavor [31]. We observed that there were no differences in the amounts of these classes of aroma compounds between two castration groups (p>0.05). Overall, the castration method showed a negligible effect on the volatile compounds in the cooked meat, this could be since the half-castration did not cause much change in the flavor precursors such as fatty acids (Table 3) or FAAs (Table 4).

**Figure 1:**
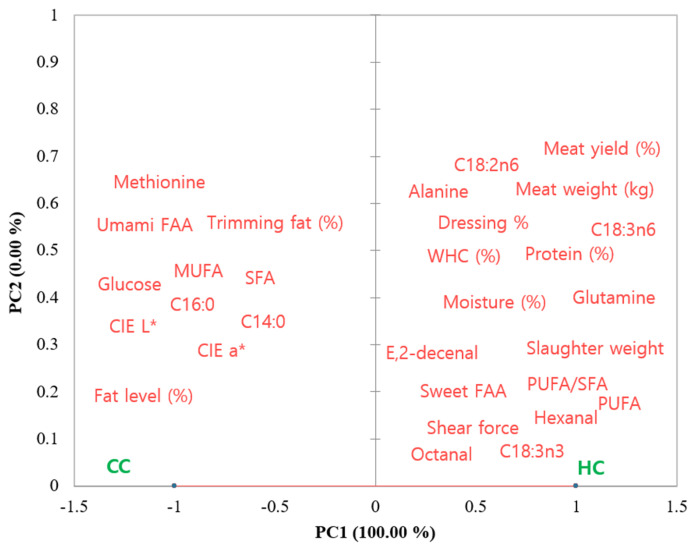
Principal component (PC) analysis for the variables (meat yield and selected quality traits) of meat from the two castration groups. The projection of variables and castration groups (CC, complete-castration; HC, half-castration) in plane defined by the PC1 and PC2.

To obtain a trend of the relationship between the castration methods and selected variables, the PCA was carried out. The PCA result (Figure 1) showed that 100% of the variability was explained by the PC1. The CC group was on the negative PC1 axis; therefore, they were related to the variables such as; total trimming fat (%), fat content, SFA, glucose, L* (lightness). Whereas, the HC was on the positive PC1 axis, therefore, they were related to total meat yield (%) and meat weight (kg), hexanal, octanal, sweet FAAs, PUFA, and PUFA/SFA etc. This PCA again indicates the differences in the meat yield and some quality traits between the two castration groups. These observations were also similar to the results presented in Table 1 to 5.

## CONCLUSION

This study for the first time evaluated the effects of castration methods (half-castration and complete castration) on the meat yield and quality characteristics of male cattle. Compared to the complete castration (steers), the half-castration produced more meat yield in almost primal cuts. Compared to the intact males, the fat content in both muscles from the half-castrated animals increased double. More importantly, the half-castration improved the meat tenderness by reducing the shear force values. The half-castration did not affect the MUFAs content, but it increased the total PUFAs content. The total amount of FAAs associated with sweet taste was found to be higher in both muscles from half-castrated animals. Also, the castration method only exhibited a negligible effect on metabolites and volatile aroma compounds in the cooked meat. Based on the results obtained from this study, it may be concluded that the half-castration could be considered as an effective solution to be used for increasing the meat yield and tenderness of male cattle.

## Figures and Tables

**Table 1:** Carcass traits and meat yield of primal cuts of Hanwoo cattle as affected by castration method

Items	Complete castration	Half-castration	Complete castration	Half-castration	Complete castration	Half-castration	Complete castration	Half-castration
Slaughter and carcass weight								
Slaughter weight (kg)	678.40±58.16	702.20±39.12						
Hot carcass weight (kg)	421.18±46.15	433.22±24.47						
Cold carcass weight (kg)	418.08±45.99	434.35±23.87						
Dressing percentage (%)	62.58±1.14	62.85±1.56						
		
	**Meat yield**				**Trimmed fat yield**			
		
Primal cuts	**Weight (kg)**		**Percentage (%)**		**Weight (kg)**		**Percentage (%)**	
		
Tenderloin	6.65±0.78^b^	7.58±0.59^a^	1.59±0.15^b^	1.75±0.13^a^	2.76±0.65	2.56±0.54	0.66±0.14	0.59±0.11
Loin	32.36±3.34^b^	38.11±3.33^a^	7.76±0.46^b^	8.77±0.60^a^	7.85±1.65^a^	4.65±1.11^b^	1.87±0.28^a^	1.07±0.24^b^
Striploin	9.38±0.95^b^	10.40±0.69^a^	2.25±0.20^b^	2.39±0.11^a^	3.46±0.78^a^	2.17±0.82^b^	0.83±0.16^a^	0.50±0.18^b^
Chuck roll	18.49±2.93^b^	28.61±3.56^a^	4.42±0.50^b^	6.59±0.77^a^	2.68±0.87^a^	1.81±0.57^b^	0.64±0.20^a^	0.42±0.12^b^
Clod	25.22±2.53^b^	30.40±2.18^a^	6.05±0.35^b^	7.01±0.49^a^	8.40±1.68^a^	6.12±1.38^b^	2.01±0.34^a^	1.41±0.30^b^
Top round	21.80±2.09^b^	27.06±1.75^a^	5.24±0.51^b^	6.24±0.39^a^	5.16±1.33^a^	4.24±1.10^b^	1.22±0.24^a^	0.97±0.22^b^
Bottom round	35.63±3.87^b^	41.23±3.13^a^	8.54±0.66^b^	9.50±0.58^a^	9.70±1.86^a^	6.95±1.71^b^	2.30±0.25^a^	1.60±0.37^b^
Brisket	43.29±6.16^b^	49.91±4.46^a^	10.34±0.63^b^	11.49±0.70^a^	54.09±10.04^a^	37.12±7.68^b^	12.89±1.57^a^	8.52±1.55^b^
Shank	15.88±1.61^b^	17.91±1.40^a^	3.81±0.27^b^	4.13±0.28^a^	3.55±0.64^a^	2.89±0.64^b^	0.85±0.13^a^	0.66±0.13^b^
Rib	50.61±5.79	52.10±3.13	12.11±0.46	12.01±0.61	11.88±3.47^a^	6.77±1.96^b^	2.81±0.60^a^	1.55±0.42^b^
Total meat/fat yield	259.30±3.05^b^	303.32±2.42^a^	62.11±0.41^b^	67.88±0.46^a^	109.53±2.29^a^	75.28±1.75^b^	26.08±0.39^a^	17.29±0.36^b^

a,b Means within a same row in each parameter with different superscripts are significantly different (p<0.05).

**Table 2:** Chemical composition and technological quality traits of *L. lumborum* and *semimembranosus* muscles as affected by castration method

Items	*L. lumborum*			*S. semimembranosus*		
	
	Intact males^1)^	Complete castration	Half-castration	Intact males^1)^	Complete castration	Half-castration
Chemical composition						
Moisture (%)	70.21±2.12^a^	62.10±4.24^c^	67.21±3.21^b^	71.38±1.54^a^	67.21±3.21^b^	70.43±2.02^a^
Fat (%)	4.20±1.48^c^	16.45±6.62^a^	8.02±4.03^b^	2.49±0.97^c^	8.02±4.03^a^	4.28±2.39^b^
Protein (%)	21.30±1.55^a^	18.75±1.57^c^	20.70±0.92^b^	21.58±1.61^a^	20.07±0.92^c^	21.11±0.69^b^
Technological quality traits						
pH	5.82±0.11^a^	5.54±0.01^b^	5.53±0.02^b^	5.89±0.05^a^	5.50±0.03^b^	5.49±0.02^b^
WHC (%)	55.25±5.34^a^	46.67±4.44^b^	46.00±5.85^b^	53.89±6.43^a^	45.77±4.74^b^	45.83±4.98^b^
Shear force (kg)	7.72±1.17^a^	5.54±1.20^b^	5.68±1.03^b^	7.02±1.32^a^	5.17±1.17^b^	5.92±1.05^c^
L* (Lightness)	33.53±3.70^c^	39.89±4.35^a^	36.50±2.50^b^	34.11±4.39^b^	37.70±3.28^a^	36.23±2.68^ab^
a* (Redness)	18.32±2.22^a^	17.44±2.03^b^	16.28±2.24^c^	22.09±4.49^a^	18.37±2.62^b^	17.86±2.12^b^
b* (Yellowness)	7.94±1.33^b^	8.34±1.28^a^	7.46±1.07^b^	10.23±2.97^a^	8.81±1.82^b^	8.32±1.34^b^

WHC, water holding capacity.

1) The data used in the statistical model was obtained from the same muscles at same post-slaughter period (24 h) of intact males (harvested at 25.74-months of age, body weight of 728 kg, raised under identical conditions) of the same cattle breed (Hanwoo) in another of our research projects (Project No. PJ01212501).

a–c Means within a same row in each muscle with different superscripts are significantly different (p<0.05).

**Table 3:** Fatty acid profiles (% of total fatty acids) of *L. lumborum* and *semimembranosus* muscles as affected by castration method

Item	*L. lumborum*		*M. semimembranosus*	
	
	Half-castration	Complete castration	Half-castration	Complete castration
C14:0	2.63±0.47^b^	3.17±0.69^a^	2.68±1.49	2.86±0.69
C16:0	26.94±1.92^b^	28.83±1.84^a^	30.38±10.19	30.87±2.93
C16:1n7	3.24±0.71	3.59±0.64	3.24±1.51	3.59±0.84
C18:0	12.86±1.81	11.54±1.41	13.47±3.03	11.92±1.54
C18:1n9	50.82±3.55	50.50±2.23	51.91±16.12	48.15±3.98
C18:1n7	0.31±0.11	0.40±0.14	0.21±0.09^b^	0.34±0.14^a^
C18:2n6	2.65±1.03^a^	1.65±0.31^b^	4.44±2.57^a^	1.89±0.29^b^
C18:3n6	0.02±0.01^a^	0.01±0.01^b^	0.01±0.01	0.01±0.00
C18:3n3	0.06±0.02^a^	0.03±0.01^b^	0.09±0.03^a^	0.03±0.01^b^
C20:1n9	0.23±0.12	0.18±0.08	0.17±0.08	0.16±0.06
C20:4n6	0.21±0.28	0.07±0.03	0.79±0.84^a^	0.15±0.07^b^
C20:5n3	0.00±0.01	0.00±0.00	0.01±0.02	nd
C22:4n6	0.03±0.02	0.02±0.01	0.07±0.05	0.03±0.01
SFA	42.43±3.58	43.55±1.75	43.24±3.66	45.65±3.03
UFA	57.57±3.58	56.45±1.75	56.76±3.66	54.35±3.03
MUFA	54.60±3.68	54.67±1.89	51.51±3.44	52.24±3.21
PUFA	2.97±1.32^a^	1.78±0.34^b^	5.25±3.56^a^	2.11±0.32^b^
n3	0.06±0.02^a^	0.03±0.01^b^	0.09±0.05^a^	0.03±0.01^b^
n6	2.91±1.30^a^	1.75±0.33^b^	5.16±3.52^a^	2.08±0.31^b^
n6/n3	51.70±10.95	60.27±19.13	53.25±14.32	63.47±16.97
MUFA/SFA	1.30±0.19	1.26±0.10	1.20±0.16	1.15±0.16
PUFA/SFA	0.07±0.03^a^	0.04±0.01^b^	0.13±0.10^a^	0.05±0.01^b^

nd, not detectable; SFA, saturated fatty acid; UFA, unsaturated fatty acid; MUFA, monounsaturated fatty acid; PUFA, polyunsaturated fatty acid.

a,b Means within a same row in each muscle with different superscripts are significantly different (p<0.05).

**Table 4:** Concentration (mg/100 g) of free amino acids of *L. lumborum* and *semimembranosus* muscles as affected by castration method

Items	*L. lumborum*		*M. semimembranosus*	
	
	Half-castration	Complete castration	Half-castration	Complete castration
Glycine	0.24±0.44	0.26±0.47	0.31±0.61	0.47±0.87
Alanine	24.16±4.29^a^	20.45±3.62^b^	21.80±6.23	20.83±6.04
Serine	2.61±0.56	2.44±0.70	2.31±0.70	2.46±0.53
Proline	1.47±0.49	1.24±0.53	1.20±0.56	1.24±0.53
Valine	3.25±0.88	2.99±0.94	3.58±1.37	3.95±1.27
Threonine	2.00±0.55	1.91±0.67	1.91±0.70	2.14±0.67
Leucine	4.81±1.37	4.82±1.49	5.67±2.43	6.84±1.96
Isoleucine	1.73±0.55	1.79±0.70	2.06±0.85	2.54±0.97
Aspartate	0.62±0.24	0.58±0.24	0.57±0.22	0.55±0.23
Lysine	3.82±0.75	3.80±0.97	4.00±1.03	4.31±1.24
Glutamate	2.53±0.94	3.02±1.38	3.71±2.18	4.07±1.34
Methionine	0.16±0.27^b^	0.43±0.33^a^	0.36±0.39^b^	0.76±0.39^a^
Histidine	2.26±0.49	2.09±0.50	2.34±0.61	2.27±0.60
Phenylalanine	1.87±0.70	2.00±0.71	2.31±1.29	2.99±0.97
Arginine	4.88±0.94	4.44±1.09	4.65±1.35	4.97±1.31
Tyrosine	2.42±0.51	2.38±0.63	2.51±0.91	2.94±0.63
Cysteine	0.24±0.00	0.24±0.00	0.24±0.00	0.24±0.00
Asparagine	0.85±0.18	0.93±0.17	0.83±0.20	0.86±0.20
Glutamine	17.27±4.24^a^	13.34±4.49^b^	12.04±3.94^a^	8.33±1.55^b^
Total FAAs associated with sweetness	47.91±7.17^a^	40.07±7.67^b^	39.93±10.80	36.24±7.84
Total FAAs associated with umami	3.16±1.06	3.60±1.42	4.28±2.33	4.62±1.47
Total FAAs associated with sourness	25.04±5.43	24.32±6.54	27.12±9.39	30.82±8.50

SM, semimembranosus; FAAs, free amino acids.

a,b Means within a same row in each muscle with different superscripts are significantly different (p<0.05).

**Table 5:** Metabolomics profiles (% of total metabolites) in *L. lumborum* and *Semimembranosus* muscles as affected by castration method

Metabolites	*L. lumborum*		*M. semimembranosus*	
	
	Complete castration	Half-castration	Complete castration	Half-castration
Acetate	0.45±0.13	0.60±0.11	0.56±0.09	0.69±0.23
Alanine	1.66±0.12	1.58±0.23	1.69±0.35	1.74±0.31
Anserine	1.81±0.24	2.26±0.57	2.39±0.19	2.36±0.18
Betaine	0.51±0.17	0.45±0.06	0.45±0.17	0.41±0.10
Carnitine	2.05±0.21	1.81±0.52	1.85±0.19	1.77±0.53
Creatine	18.36±0.95	18.68±0.66	17.17±0.72	17.52±1.26
Creatinine	0.67±0.08	0.69±0.14	0.79±0.21	0.78±0.18
Fumarate	0.23±0.09	0.46±0.29	0.32±0.19	0.26±0.27
Glucose	2.47±0.44^a^	1.54±0.50^b^	2.01±0.61	2.04±0.52
Glucose-6-phosphate	3.45±0.68	2.69±0.69	3.15±0.59	3.07±0.72
Glutamine	1.05±0.38	1.47±0.28	0.89±0.17	1.23±0.37
Glutathione	0.28±0.12	0.26±0.07	0.24±0.11	0.25±0.01
Glycerol	2.95±0.37	3.34±0.41	3.33±0.24	3.48±0.41
Glycine	1.35±0.20	1.44±0.24	1.42±0.23	1.42±0.23
IMP	1.25±0.54	1.33±0.30	1.39±0.71	0.81±0.39
Inosine	0.46±0.17	0.59±0.19	0.53±0.32	0.53±0.26
Isoleucine	0.19±0.02	0.18±0.02	0.23±0.05	0.20±0.04
Lactate	56.71±1.62	56.38±1.43	57.52±0.79	57.29±1.31
Leucine	0.38±0.06	0.33±0.03	0.41±0.06	0.32±0.07
Malonate	0.54±0.10	0.56±0.15	0.53±0.12	0.54±0.10
Methionine	0.15±0.03b	0.21±0.02^a^	0.16±0.06	0.18±0.08
N,N-Dimethylglycine	0.06±0.00	0.07±0.00	0.06±0.00	0.06±0.00
N-Nitrosodimethylamine	0.22±0.02	0.21±0.02	0.21±0.02	0.21±0.01
Niacinamide	0.27±0.04	0.31±0.06	0.27±0.04	0.27±0.03
O-Acetylcarnitine	0.70±0.09	0.77±0.16	0.76±0.05	0.72±0.20
Pyruvate	0.14±0.18	0.09±0.08	0.05±0.02	0.05±0.01
Succinate	0.48±0.47	0.37±0.31	0.48±0.34	0.47±0.39
Tyrosine	0.13±0.01	0.14±0.05	0.13±0.02	0.14±0.03
Valine	0.26±0.03	0.25±0.04	0.29±0.02	0.26±0.08
sn-Glycero-3-phosphocholine	0.76±0.16	0.92±0.35	0.74±0.16^b^	0.94±0.11^a^

a,b Means within a same row with different superscripts are significantly different (p<0.05).

**Table 6:** Concentration (μg/g) of volatile flavor compounds in *L. lumborum* muscles as affected by castration method

Compounds	Retention time (min)	IM^1)^	Complete castration	Half-castration
Aldehydes				
2-methyl pentanal	1.6608	MS, STD	0.017±0.01	0.026±0.02
2-methyl propanal	1.8778	MS, STD	0.006±0.00	0.010±0.01
3-methyl butanal	2.587	MS, STD	0.014±0.01	0.017±0.01
2-methyl butanal	2.699	MS, STD	0.014±0.01	0.016±0.01
Pentanal	3.154	MS, STD	0.076±0.03	0.134±0.12
Heptanal	8.9842	MS, STD	0.090±0.05	0.148±0.09
Hexanal	5.8122	MS, STD	1.440±0.62^b^	2.666±1.82^a^
Benzaldehyde	10.541	MS, STD	0.156±0.19	0.181±0.16
Octanal	11.6266	MS, STD	0.135±0.05^b^	0.194±0.17^a^
Nonanal	13.8771	MS, STD	0.071±0.03	0.107±0.05
E,2-nonenal	14.9997	MS, STD	0.030±0.02	0.021±0.01
Benzeneacetaldehyde	12.5696	MS, STD	0.007±0.00	0.008±0.00
E,2-Octenal	12.8868	MS, STD	0.005±0.00	0.010±0.01
Decanal	15.8893	MS, STD	0.019±0.02	0.079±0.08
E,2-decenal	16.9166	MS, STD	0.015±0.00^b^	0.023±0.01^a^
Undecenal	17.721	MS, STD	nd	0.004±0.00
2-undecenal	18.69	MS, STD	0.003±0.00	0.003±0.00
Alcohols				
2-Heptanol	3.615	MS, STD	0.008±0.01	0.011±0.02
1-Pentanol	4.7849	MS, STD	0.041±0.02	0.082±0.06
Hexanol	5.526	MS, STD	0.004±0.00	0.008±0.01
1-Hexanol	8.0362	MS, STD	0.019±0.01	0.025±0.02
1-Heptanol	10.827	MS, STD	0.033±0.03	0.053±0.05
1-Octen-3-ol	11.097	MS, STD	0.017±0.01^b^	0.043±0.03^a^
1-Octanol	13.1569	MS, STD	0.004±0.00	0.007±0.01
Sulfur- and nitrogen-containing compounds				
Methanethiol	1.5234	MS, STD	0.003±0.00	0.004±0.00
Carbon disulfide	1.7352	MS, STD	0.003±0.00	0.005±0.00
Methional	9.0956	MS, STD	0.004±0.00	nd
Benzothiazole	16.297	MS, STD	nd	0.006±0.00
Pyrazines				
Methylpyrazine	6.554	MS, STD	0.014±0.02	0.015±0.02
2,5-dimethylpyrazine	9.1856	MS, STD	0.026±0.01	0.037±0.03
2,3-dimethylpyrazine	9.4184	MS	0.029±0.00	0.009±0.01
3-Ethyl-2,5-dimethylpyrazine	13.3741	MS	0.008±0.00	0.013±0.01
2-ethyl-3,5-dimethyl pyrazine	13.4569	MS	0.003±0.00	nd
Hydrocarbons				
Toluene	4.7161	MS, STD	0.004±0.00	0.006±0.00
Ethylbenzene	7.7503	MS, STD	0.003±0.00	0.006±0.00
1,3-dimethylbenzene	7.9939	MS, STD	0.026±0.02	0.044±0.03
2(5H)-Furanone	9.365	MS	0.026±0.01	0.028±0.03
5-ethyl-2-methyloctane	12.3414	MS	0.009±0.01	0.011±0.01
2,6-dimethyloctane	12.7915	MS	0.008±0.00	0.011±0.01
2,5,9-trimethyldecane	13.0034	MS	0.005±0.00	0.007±0.00
4,8-dimethyldecane	13.2628	MS	0.008±0.00	0.007±0.00
Octanoic acid	15.148	MS, STD	0.037±0.03	0.022±0.01
Dodecane	15.7622	MS, STD	0.049±0.01^b^	0.100±0.06^a^
Anethole	17.4938	MS	0.007±0.00	0.021±0.02
Tetredecane	19.262	MS, STD	0.005±0.00	0.010±0.01

IM, identification method; nd, not detectable.

1) The compounds were identified by mass spectra (MS) from library or external standards (STD).

a,b Means within a same row with different superscripts are significantly different (p<0.05).
